# Disparities in HPV vaccine knowledge and adolescent HPV vaccine uptake by parental nativity among diverse multiethnic parents in New Jersey

**DOI:** 10.1186/s12889-022-12573-7

**Published:** 2022-01-29

**Authors:** Bianca Anuforo, Jennifer K. McGee-Avila, Lindsey Toler, Baichen Xu, Racquel E. Kohler, Sharon Manne, Jennifer Tsui

**Affiliations:** 1 Cancer Institute of New Jersey, Rutgers, The State University of New Jersey, New Brunswick, NJ USA; 2 School of Nursing, Rutgers, The State University of New Jersey, Newark, NJ USA; 3 School of Public Health, Rutgers, The State University of New Jersey, Piscataway, NJ USA; 4 Department of Population and Public Health Sciences, Keck School of Medicine, University of Southern California, 1441 Eastlake Ave, Los Angeles, CA 90033 USA

**Keywords:** HPV vaccine, Parental knowledge, Immigrant populations, Minority health, Vaccine uptake

## Abstract

**Background:**

Suboptimal human papillomavirus (HPV) vaccination rates persist among adolescents in the United States (U.S.). New Jersey (NJ), among the top, most racially/ethnically diverse states in the U.S., had among the lowest HPV vaccine initiation rates, prior to 2018. This study examined parental HPV vaccine knowledge and adolescent HPV vaccine initiation among multiethnic parents in NJ, where access to language concordant HPV vaccine information and vaccination services may differ, for immigrant parents.

**Methods:**

We surveyed parents of adolescents (ages 11–18) at community events in NJ to examine parental HPV vaccine knowledge and adolescent HPV vaccine uptake. Vaccine knowledge was assessed using an 11-item question stem that covered vaccine efficacy, gender recommendation, vaccine protection, and myths. Multivariable models assessed the association of parent nativity on HPV vaccine knowledge scores and adolescent HPV vaccine initiation, controlling for sociodemographic factors.

**Results:**

Of the 77 parents, most parents (84%) were aware of the HPV vaccine. However, knowledge scores were low and differed by parent nativity. Non-U.S. born parents had significantly lower knowledge scores − 1.7 [− 3.1, − 0.4] and lower odds of adolescent children initiating the HPV vaccine 0.3 [0.1, 0.9] compared to U.S.-born parents after adjusting demographic characteristics.

**Conclusions:**

Our findings reveal that parental HPV vaccine knowledge remains low among suburban dwelling, immigrant parents, even though they have higher education and access to health care. Multilevel strategies to reduce missed opportunities for HPV vaccine education among parents and HPV vaccination for adolescents are needed, including for suburban, immigrant communities.

**Supplementary Information:**

The online version contains supplementary material available at 10.1186/s12889-022-12573-7.

## Introduction

Although human papillomavirus (HPV) vaccines prevent infection of high-risk HPV types associated with multiple cancers, disparities in incidence of cervical cancer and other HPV-associated cancers continue to persist among racial/ethnic minority and immigrant populations [1]. Routine HPV vaccination has been recommended in the United States (U.S.) for adolescent girls since 2006 and adolescent boys since 2011. However, uptake continues to be suboptimal at only 71.5% initiation among adolescents ages 13–17 years old [2]. Lower rates of HPV vaccine uptake are observed among adolescents of immigrant parents [3, 4]. Prior studies have attributed lower HPV vaccination rates among adolescents of immigrant parents to lack of strong provider recommendation [5], lack of perceived benefits of the vaccine, concerns about sexual activity [6], and limited access to vaccine information. However, few studies have focused on suburban dwelling, immigrant parents where socioeconomic status and access to health care are on average higher than racial/ethnic minority and immigrant parents in urban communities, but HPV vaccination rates remain low in many communities.

HPV vaccine knowledge among parents continues to be an important predictor of HPV vaccine initiation [7]. Despite increasing numbers of immigrants in the U.S. [8], assessments of HPV vaccine knowledge among immigrants parents have primarily focused on Latinx/Hispanic groups, parents of low socioeconomic status, and other medically underserved populations [9, 10]. Studies have shown that immigrant parents often have little knowledge of the HPV vaccine and vaccine decision-making is often influenced by cultural norms and sexual misconceptions [11, 12]. It is unclear whether access to HPV vaccine information and limited knowledge about HPV vaccines persists as a key barrier to vaccine uptake among immigrant parents living in surburban, higher socioeconomic areas. Prior studies in suburban communities have shown that while these areas have higher unadjusted access to health care overall, a large proportion of surburban residents lack access to health care and remain medically vulnerable [13, 14]. Studying this population may provide a better understanding of why HPV vaccine knowledge and HPV vaccine uptake remain lower in New Jersey compared to neighboring metropolitan areas such as Philadelphia and New York City [15] despite having higher education and socioeconomic status at the population level.

This study examines parental HPV vaccine knowledge and adolescent HPV vaccine initiation among multiethnic parents in New Jersey, where access to language concordant HPV vaccine information and vaccination services may differ from their urban counterparts, particularly for immigrant parents. New Jersey has higher than average HPV-associated cancer incidence [16] and low HPV vaccine initiation rates as well as immense population-level diversity in race/ethnicity and birthplace. We assess whether HPV vaccine knowledge among parents and HPV vaccine initiation among adolescents differ by parental nativity to inform local level needs for HPV vaccine education and promotion. We hypothesize that of our New Jersey study sample, non-U.S. born parents will have lower HPV vaccine knowledge compared to U.S.-born parents and adolescents of non-U.S. born parents will have lower HPV vaccine initiation compared adolescents of U.S.-born parents.

## Methods

### Study procedures

We conducted a cross-sectional study to evaluate HPV vaccine knowledge among parents and examine parental report of HPV vaccine initiation among their adolescents in a diverse sample of multiethnic parents in New Jersey. Parents were recruited from 14 cancer prevention and control events, including health fairs, community events, middle/high school parent meetings, and cancer forums held by the Rutgers Cancer Institute of New Jersey Community Outreach and Engagement and other community partners between October 2018 and March 2020. Eligible survey participants included parents who 1) were able to read, speak and write in English, Spanish or Traditional Chinese, 2) had an adolescent between the ages of 11–18 years in their household; and 3) were the health care decision maker for the adolescent(s). All adult event attendees in proximity to research staff during community events were approached, asked about their interest in participating in the study and assessed for eligibility. All participants provided verbal consent prior to survey administration. Participants were given a study information sheet detailing the study and their rights as a participant. Upon survey completion, parents were given an HPV vaccine fact sheet from the American Cancer Society titled, *Protecting Our Children from HPV Cancer* in their preferred language (i.e. English, Chinese or Spanish). Participants who completed at least 75% of the survey received a $10 Target gift card. The research study protocol was approved by the Rutgers New Brunswick Health Sciences Institutional Review Board.

### Survey instrument

The self-administered, 42-item paper survey collected information on HPV vaccine awareness and knowledge, access to and uptake of the HPV vaccine, receipt of other adolescent immunizations and parent and adolescent sociodemographic characteristics. The survey was developed for this study (see Additional file 1). Questions were adapted from the Health Information National Trends Survey, the National Immunization Survey (Teen data) as well as prior work of the research team [17]. The survey items included questions from national and state-based population level surveys in order to capture responses by groups unable to complete the survey in their native language. Surveys were administered to parents in the following languages: English, Traditional Chinese, Korean and Spanish. Additionally, the languages selected are largely spoken by non-English speaking groups and relflect the diversity of languages in New Jersey [18]. All research study and recruitment materials were translated by an external translation service that specializes in cultural adaptation; back translations were reviewed by native speakers affiliated with our research team to ensure accuracy and appropriate reading level. Surveys were completed by parents during the in-person community events and took approximately 10–15 min to complete.

### Measures

#### HPV and HPV vaccine knowledge

The primary outcomes of interest were: 1) HPV vaccine knowledge and 2) adolescent HPV vaccine initiation. HPV vaccine knowledge was assessed in an 11-part question that covered vaccine efficacy, gender recommendation, dosage, benefits, side effects, vaccine protection, and myths about the HPV vaccine as used in prior studies [17]. We examined individual knowledge items as well as comparing the HPV vaccine belief items (items 1–3) from HPV knowledge items (4–11) separately, and then summed correct responses to the 11 items into a composite HPV vaccine knowledge score assigning, “don’t know,” or “not sure” responses as incorrect. The composite HPV vaccine knowledge score ranged from 0 to 11.

#### HPV vaccine initiation

HPV vaccine initiation was assessed by asking parents whether their adolescent child ever received the HPV vaccine or HPV shot (yes or no). Parents who reported having multiple adolescent children within the eligible age range were asked to report HPV vaccination history about the child with the closest birthday (month/day) to the survey administration date as an attempt to vary age ranges of HPV vaccine eligible adolescents in our sample.

#### Nativity

The primary independent variable of interest was parent nativity. Parent nativity was assessed by the following question: Were you born in the United States? (yes, no, or prefer not to answer). Parents who preferred not to answer or did not answer were dropped from the analysis.

#### Sociodemographic information

Parent sociodemographic characteristics included age, gender, race/ethnicity (Non-Hispanic Black, Non-Hispanic White, Hispanic, Non-Hispanic Asian and multi-race), education (no college degree, college degree or graduate degree) and annual household income.

Adolescent sociodemographic variables included age, sex (male or female), and insurance type (i.e., private insurance, Medicaid/CHIP or other/uninsured). We categorized adolescent age based on the recommended age to begin the HPV vaccine series (i.e., 11–12, 13–15, and 16–18 years).

#### Other adolescent vaccinations

In addition to HPV vaccination, parents were asked about receipt of other adolescent vaccinations (Tdap and meningococcal vaccines). Parents who answered “I don’t know” were combined with “no” category. Vaccination was assessed individually and by a binary measure indicating receipt of at least one of the other two adolescent vaccines.

### Statistical analysis

Descriptive statistics were computed for independent variables of interest among both parents and adolescents and to assess their relationship with HPV vaccine knowledge and initiation. Of the 324 individuals approached by research staff at community events, 125 individuals were eligible to participate and 110 participants completed the survey. In our final analytic sample, we dropped an additional 17 parents due to missing sociodemographic information in our final models. The final analytic sample included 77 parents, after excluding participants with missing data on adolescent vaccination status or nativity. Generalized linear model assessed relationships between sociodemographic characteristics (parents and adolescents) and parental HPV vaccine knowledge composite scores. We performed a sensitivity analysis to examine if HPV vaccine belief items (items 1–3) differed significantly from HPV knowledge items (4–11) from the composite knowledge measure. Beta (β) coefficients and 95% confidence intervals (95% CI), are presented. Logistic regression models assessed relationships between sociodemographic characteristics (parents and adolescents) and parent reported HPV vaccine initiation for their adolescent children. Odds ratios and 95% CIs are reported. All analyses were conducted using SAS version 9 [19].

## Results

### Sample characteristics

Of the 77 parents included in our analysis, 40% were non-U.S. born (Table 1). The study sample included mostly mothers (84%), who were non-Hispanic (NH)-Asian (26%), Hispanic (22%), or NH-white (30%). Most parents had at least a college degree (74%), were privately insured (77%), and earned at least $80,000 annually (53%). More than half (65%) of parents reported having an adolescent daughter. The mean adolescent age was 14 years. We found notable differences in distributions across sociodemographic parent and adolescent characteristics by parental nativity. Among non-U.S. born parents, a high proportion were NH-Asian (61.3%) and over half held graduate level degrees (51.6%).

**Table 1:** Sociodemographic characteristics of parents and adolescents by parent nativity (*n* = 77)

	Total *n* = 77	U.S.-Born *n* = 46	Non-U.S. Born *n* = 31	*P*-value
	%	%	%	
**Parent Characteristics**				
**Race/Ethnicity**				
Hispanic	22.1	26.1	16.1	**< 0.01**
NH-White	29.9	41.3	12.9	
NH-Black	18.2	28.3	3.2	
NH-Asian	26.0	2.2	61.3	
Other/Multi-Race/Missing	3.9	2.2	6.5	
**Survey Language**				
English	85.7	97.8	67.7	**< 0.01**
Chinese	10.4	0.0	25.8	
Spanish	3.9	2.2	6.5	
**Age**				
30–40 years	20.8	21.7	19.4	0.96
41–50 years	53.3	54.8	52.2	
> 50 years	26.0	25.8	26.1	
**Gender**				
Male	14.3	8.7	22.6	0.18
Female	84.4	89.1	77.4	
**Insurance Type**				
Private Health Insurance	76.6	76.1	77.4	0.17
Medicaid/Medicare	13.0	17.4	6.5	
Uninsured/Other	6.5	2.2	12.9	
**Education**				
Less Than College	26.0	32.6	16.1	0.08
College Graduate	36.4	39.1	32.3	
Graduate or Higher Degree	37.7	28.3	51.6	
**Income**				
$0 - $39,999	22.1	23.9	19.4	0.74
$40,000 - $79,999	15.6	17.4	12.9	
> $80,000	53.3	52.2	54.8	
Missing	9.1	6.5	12.9	
**Adolescent Characteristics**				
**Age**				
11–12 years	23.4	26.1	19.4	0.48
13–15 years	40.3	34.8	48.4	
16–18 years	36.3	39.1	32.3	
**Gender**				
Male	35.1	43.5	22.6	0.06
Female	64.9	56.5	77.4	
**Insurance Type**				
Private	76.6	76.1	77.4	0.23
Medicaid/CHIP	15.6	19.6	9.7	
Uninsured/Other/Missing	7.8	4.4	12.9	
**HPV Vaccine Initiation**				
Yes	57.1	52.2	29.0	**0.04**
No	42.9	47.8	71.0	
**Other Adolescent Vaccinations**				
Yes	45.5	60.9	45.2	0.16
No	54.6	39.1	54.8	

*NH* Non-Hispanic, *CHIP* Children’s Health Insurance Program

### HPV vaccine knowledge

In our sample, 84% of parents were aware of the HPV vaccine. Parents were least knowledgeable about the protection from genital warts or the importance of being vaccinated before sexual debut. HPV vaccine knowledge differed by nativity (Table 2). When compared to U.S.-born parents, specific differences in knowledge were also observed. For example, a lower proportion of non-U.S. born parents (54.8%) knew HPV vaccines protect against cervical cancer compared to U.S.-born parents (76.1%) (*p* < 0.05). Additionally, a much lower proportion of non-U.S. born parents knew that the HPV vaccine is recommended for both boys and girls (non-U.S. born parents, 32.3% vs U.S.-born parents 69.6%, *p* < 0.01). We did not observe significant findings in our sensitivity analysis of examining HPV belief questions (items 1–3) and HPV knowledge questions (items 4–11). Thus, we kept all 11 knowledge items in the composite HPV knowledge score and report those findings in the remaining analyses.

**Table 2:** HPV vaccine knowledge among parents of vaccine eligible adolescents in New Jersey (*n* = 77)

Statement	U.S.-Born		Non-U.S. Born		*P*-value
	Incorrect/Not sure		Incorrect/Not sure		
	n	%	n	%	
1.HPV^a^ vaccine may cause problems getting pregnant/conceiving a child	21	46.7	17	54.8	0.43
2. HPV vaccine may cause health problems in the future	22	47.8	19	61.3	0.25
3. My child may be more likely to think it is okay to have sex if he/she gets the HPV vaccine	17	37.0	16	51.6	0.40
4. HPV vaccines require more than one dose	18	39.1	19	61.3	0.06
5. HPV vaccines offer protection against all STIs^b^	7	15.2	14	45.2	**< 0.01**
6. HPV vaccines are most effective if given to people who have never had sex	22	47.8	23	74.2	0.06
7. Someone who has had the HPV vaccine cannot develop cervical cancer	11	23.9	14	45.2	**0.05**
8. Protection against most cancers	36	78.3	27	87.1	0.32
9. One of the HPV vaccines offers protection against genital warts	34	73.9	20	64.5	0.38
10. HPV vaccinated girls do not need a Pap smear test	5	10.9	12	38.7	**< 0.01**
11. HPV vaccine is only recommended for girls	8	17.4	18	58.1	**< 0.01**

Response categories for items 1 through 3 were: Agree, disagree or not sure. Response categories for items 4 through 11 were: true, false or do not know

^a^ *HPV* Human papillomavirus, ^b^ *STI* Sexually transmitted infections

Table 3 shows the unadjusted and adjusted results for both HPV vaccine knowledge and initiation from our linear regression models. Unadjusted models showed U.S-born, younger parents, private insurance, higher income parents had significantly higher HPV vaccine knowledge score compared to their counterparts. In adjusted models, the relationship between nativity and HPV vaccine knowledge remained significant after controlling for other parent and adolescent factors, where non-U.S.-born parents had on average − 1.7 (95% CI: − 3.1, − 0.4) lower HPV vaccine knowledge score compared to U.S. born parents, after controlling for parent nativity and age and adolescent age and insurance type.

**Table 3:** Unadjusted and adjusted models for HPV vaccine knowledge score and HPV vaccine initiation (*n* = 77)

	HPV vaccine knowledge		HPV vaccine initiation	
	Unadjusted B [95% CI]	Adjusted B [95% CI]	Unadjusted OR [95% CI]	Adjusted OR [95% CI]
**Parent Characteristics**				
Nativity				
Non-U.S. Born	−2.0 [−3.3, −0.7]	−1.7 [− 3.1, − 0.4]	0.4 [0.1, 1.0]	0.3 [0.1, 0.9]
U.S.-Born	Reference	Reference	Reference	Reference
Age (years)				
30–40	Reference	Reference	–	–
41–50	1.3 [−0.4, 3.0]	0.8 [−1.3, 2.8]	–	–
> 50	2.0 [0.1, 4.0]	1.5 [−0.7, 3.7]	–	–
HPV Vaccine Knowledge	–	–	1.3 [1.1, 1.5]	1.3 [1.0, 1.7]
**Adolescent Characteristics**				
Age (years)				
11–12	0.2 [−1.6, 2.0]	0.5 [− 1.2, 2.2]	0.02 [0.003, 0.2]	0.01 [0.002, 0.2]
13–15	−1.0 [−2.5, 0.5]	−0.5 [−2.0, 1.0]	0.3 [0.1, 1.0]	0.4 [0.1, 1.6]
16–18	Reference	Reference	Reference	Reference
Gender				
Male	–		0.3 [0.1, 0.9]	0.1 [0.0, 0.6]
Female	–			Reference
Insurance Type				
Private	Reference	Reference	–	–
Medicaid/CHIP	−0.5 [−2.3, 1.3]	−0.2 [−2.3, 2.0]	–	–
Uninsured/Other	−3.5 [−5.9, −1.1]	−2.3 [−4.9, 0.4]	–	–
Received Other Adolescent Vaccines				
Yes	–	–	3.8 [1.4, 10.1]	3.4 [0.9, 13.2]
No	–	–	Reference	Reference

“–” indicates variable excluded from final model

*HPV* Human papillomavirus vaccine, *OR* Odds ratio, *CI* Confidence interval, *CHIP* Children’s Health Insurance Program, *Tdap* Tetanus, diphtheria, and acellular pertussis

### HPV vaccine initiation

HPV vaccination initiation was low in our study sample (*n* = 57%). Only 29% of adolescents of non-U.S. born parents initiated the HPV vaccine compared to 52% adolescents of U.S.-born parents (*p* < 0.05). In the adjusted model (Table 3), adolescents of non-U.S. born parents had lower odds of initiating the HPV vaccine (adjusted odds ratio [aOR] 0.3, 95% CI:0.1,0.9) compared to adolescents of U.S.-born parents, after adjusting for parent nativity and adolescent age, gender and receipt of other adolescent vaccinations. In addition, younger adolescents had lower odds of initiating the HPV vaccine compared to older adolescents (ages 16–18 years). Higher knowledge scores were marginally associated with increased odds of HPV vaccine initiation (aOR 1.3, 95% CI: 1.0, 1.7). Additionally, we assessed the relationship between HPV vaccine initiation and receipt of other adolescent vaccinations. Overall, 54.6% of all adolescents received at least one other adolescent vaccination. Our adjusted model reveals that adolescents who received at least one other adolescent vaccine were more likely to have initiated the HPV vaccine (aOR 3.4, 95% CI: 0.9, 13.2).

## Discussion

In our study of a multiethnic sample of parents of adolescents, we observed high HPV vaccine awareness but low parental HPV vaccine knowledge and low adolescent HPV vaccine initiation. HPV vaccine knowledge was significantly lower among non-U.S. born parents compared to U.S-born parents in our study sample of parents with high educational attainment and access to health care. Our observed relationship between parental nativity and parental HPV vaccine knowledge is consistent with previous studies, conducted in immigrant populations and communities [3, 11]. Our findings on low HPV vaccine knowledge among immigrant parents in New Jersey, indicates linguistically and culturally appropriate education and outreach efforts on HPV vaccination are warranted even in higher socioeconomic areas.

HPV vaccine uptake among all adolescents was low and significantly lower among adolescent of non-U.S born parents. Less than a third of immigrant parents reported their adolescent had initiated the HPV vaccine. This is much lower compared to the 72% of adolescents who initiated the HPV vaccine series reported in 2019 National Immunization Survey-Teen data [2] and indicates the need for more granular local level assessments of HPV vaccination and barriers to uptake. As expected, we observed a significant association between HPV vaccine knowledge among parents and HPV vaccine initiation among adolescents. Consistent with literature, older adolescent age [20] was significantly associated with HPV vaccine uptake, indicating a delay by parents to get their child vaccinated despite the recommendation for younger adolescents to begin their vaccine or missed opportunities from providers to inform the parents of the vaccine. However, unlike other studies [21, 22], receipt of other adolescent vaccines was not significantly associated with HPV vaccination in our study sample, suggesting limited co-administration of HPV vaccinations with other adolescents vaccinations in our sample. Lack of co-administration of HPV vaccines with other adolescent immunizations indicate missed opportunities and the need to address other multilevel barriers to vaccination, beyond parental characteristics, at the provider, practice, and heath-system levels [23–25]. Our prior work on understanding physician perspectives in recommending the HPV vaccine reveals that parental hesistancy, coupled with lack of strong recommendations from providers are barriers to vaccine uptake. Providers interviewed indicated that co-adminstration, stronger provider recommendations and partnerships with schools would help to increase HPV vaccine uptake [26]. Ongoing efforts to improve vaccination and vaccine encounters within the health care setting should be tailored to the education and access needs of immigrant parents. These results highlight the need for a nuanced view of interventions to increase HPV vaccine uptake among adolescents, particularly among growing immigrant communities where logistical barriers to vaccine uptake and parental education may be masked by higher socioeconomic status in suburban areas. Furthermore, without policy at the population level or within health system and school-based settings, it will be difficult to reach adolescent HPV vaccination rates observed in other countries where school based vaccination programs have been shown to increase HPV vaccine uptake [27–29].

Although our study has strengths, there are some limitations. First, we focused on parents attending community outreach events led by our cancer center and community partners. While this sampling strategy may not be generalizable to parents of all adolescents in New Jersey, it did include a diverse sample of immigrant and racial/ethnic minority parents. Additionally, focusing on community outreach events may have led to a smaller sample size leading to lack of generalizability. Furthermore, the parents at the events may have already been interested in the survey topic. However, the study sample represents the target population of our cancer center catchment area for community-based cancer education and control programs and allows us to assess the needs of our community audience. In addition to our survey being translated into Spanish, our sample includes a notable proportion of Asian American parents, including those who completed the survey in Chinese, and provides insights on HPV vaccine knowledge and uptake in an otherwise understudied population. Although we had initially translated the survey into Korean, few Korean parents were in attendance at the community events we attended for study recruitment. New Jersey has the fourth largest Asian American population among states in the U.S. [30]. While our study included Chinese language survey participants, it will be important to assess HPV vaccine knowledge and initiation in other growing Asian American and Pacific Islander communities within the state in the future. Lastly, HPV vaccine initiation responses were based on parent-report and not medical records, which could lead to underreporting [31] and consequently, low vaccination uptake [32].

## Conclusions

Our study is one of the few to explore the relationship between parental nativity and HPV vaccine knowledge and adolescent HPV vaccine initiation from a diverse sample of multiethnic parents in higher socioeconomic, suburban communities. Our findings support the need to address low HPV vaccine knowledge among immigrant parents, including those in higher socioeconomic, suburban areas, where educational needs may be masked. Unique barriers, including increased distance to ethnically concordant providers and lack of targeted language concordant resources, for these suburban, immigrant communities may contribute to the persistent suboptimal rates of HPV vaccination among adolescents. Multilevel strategies to improve HPV vaccine education, address parental hesitancy, and improve access to vaccination are needed across growing immigrant communities in more suburban areas outside of inner cities.

## Supplementary Information

**Additional file 1.** NJ Healthy Families Survey English.

## Data Availability

The datasets used and/or analysed during the current study available from the corresponding author on reasonable request. The survey is available in the Additional file 1.

## Abbreviations

HPV: Human papillomavirus
US: United States
NJ: New Jersey
NH: Non-Hispanic
CHIP: Children’s Health Insurance Program
STI: Sexually transmitted infections
OR: Odds ratio
CI: Confidence interval
Tdap: Tetanus, diphtheria, and acellular pertussis

## References

[1] Bhattacharya M, Reiter PL, McRee AL (2019). Nativity status and genital HPV infection among adults in the U.S. Hum Vaccin Immunother.

[2] Elam-Evans LD, Yankey D, Singleton JA, Sterrett N, Markowitz LE, Williams CL (2020). National, regional, state, and selected local area vaccination coverage among adolescents aged 13-17 years - United States, 2019. MMWR Morb Mortal Wkly Rep.

[3] Lee YM, Riesche L, Lee H, Shim K (2018). Parental HPV knowledge and perceptions of HPV vaccines among Korean American parents. Appl Nurs Res.

[4] Zhu L, Zhai S, Siu PT, Xia HY, Lai S, Zambrano CN (2019). Factors related to Chinese parents’ HPV vaccination intention for children. Am J Health Behav.

[5] Reiter PL, Pennell ML, Martinez GA, Katz ML. Provider recommendation for HPV vaccination across Hispanic/Latinx subgroups in the United States. Hum Vaccin Immunother. 2020;17(4):1083–8. 10.1080/21645515.2020.1846399 PMC8018483 33326347

[6] Holman DM, Benard V, Roland KB, Watson M, Liddon N, Stokley S (2014). Barriers to human papillomavirus vaccination among US adolescents: a systematic review of the literature. JAMA Pediatr.

[7] Galbraith KV, Lechuga J, Jenerette CM, Moore LA, Palmer MH, Hamilton JB (2016). Parental acceptance and uptake of the HPV vaccine among African-Americans and Latinos in the United States: a literature review. Soc Sci Med.

[8] Grieco EM, Acosta Y, De La Cruz GP. The foreign-born population in the United States: 2010. US Department of Commerce, Economics and Statistics Administration, US Census Bureau; 2012. https://www2.census.gov/library/publications/2012/acs/acs-19.pdf.

[9] Glenn BA, Tsui J, Singhal R, Sanchez L, Nonzee NJ, Chang LC (2015). Factors associated with HPV awareness among mothers of low-income ethnic minority adolescent girls in Los Angeles. Vaccine.

[10] Ashing KT, Carrington A, Ragin C, Roach V (2017). Examining HPV- and HPV vaccine-related cognitions and acceptability among US-born and immigrant hispanics and US-born and immigrant non-Hispanic blacks: a preliminary catchment area study. Cancer Causes Control.

[11] Netfa F, Tashani M, Booy R, King C, Rashid H, Skinner SR (2020). Knowledge, attitudes and perceptions of immigrant parents towards human papillomavirus (HPV) vaccination: a systematic review. Trop Med Infect Dis.

[12] Aragones A, Genoff M, Gonzalez C, Shuk E, Gany F (2016). HPV vaccine and Latino immigrant parents: if they offer it, we will get it. J Immigr Minor Health.

[13] Schnake-Mahl AS, Sommers BD (2017). Health care in the suburbs: an analysis of suburban poverty and health care access. Health Aff.

[14] Tsui J, Yang A, Anuforo B, Chou J, Brogden R, Xu B (2021). Health related social needs among Chinese American primary care patients during the COVID-19 pandemic: implications for cancer screening and primary care. Front Public Health.

[15] Walker TY, Elam-Evans LD, Yankey D, Markowitz LE, Williams CL, Mbaeyi SA (2018). National, regional, state, and selected local area vaccination coverage among adolescents aged 13-17 years - United States, 2017. MMWR Morb Mortal Wkly Rep.

[16] Walker TY, Elam-Evans LD, Yankey D, Markowitz LE, Williams CL, Fredua B (2019). National, regional, state, and selected local area vaccination coverage among adolescents aged 13-17 years - United States, 2018. MMWR Morb Mortal Wkly Rep.

[17] Waller J, Ostini R, Marlow LA, McCaffery K, Zimet G (2013). Validation of a measure of knowledge about human papillomavirus (HPV) using item response theory and classical test theory. Prev Med.

[18] Health NJDo. New Jersey State Health Assessment Data PO Box 360, Trenton, NJ 08625–03602021 [updated 09/28/2021. Available from: https://www-doh.state.nj.us/doh-shad/indicator/view/Demographics.Language.html.

[19] SAS Institute Inc. SAS 9.4. SAS Campus Drive, Cary, North Carolina 27513, USA; 2002.

[20] Dela Cruz MRI, Braun KL, Tsark JAU, Albright CL, Chen JJ (2020). HPV vaccination prevalence, parental barriers and motivators to vaccinating children in Hawai’i. Ethn Health.

[21] Kepka D, Bodson J, Lai D, Sanchez-Birkhead A, Villalta J, Mukundente V (2018). Factors associated with human papillomavirus vaccination among diverse adolescents in a region with low human papillomavirus vaccination rates. Health Equity.

[22] Kessels SJ, Marshall HS, Watson M, Braunack-Mayer AJ, Reuzel R, Tooher RL (2012). Factors associated with HPV vaccine uptake in teenage girls: a systematic review. Vaccine.

[23] Taplin SH, Anhang Price R, Edwards HM, Foster MK, Breslau ES, Chollette V (2012). Introduction: understanding and influencing multilevel factors across the cancer care continuum. J Natl Cancer Inst Monogr.

[24] Lake PW, Kasting ML, Christy SM, Vadaparampil ST (2019). Provider perspectives on multilevel barriers to HPV vaccination. Hum Vaccin Immunother.

[25] Rodriguez SA, Mullen PD, Lopez DM, Savas LS, Fernández ME (2020). Factors associated with adolescent HPV vaccination in the US: a systematic review of reviews and multilevel framework to inform intervention development. Prev Med.

[26] Tsui J, Vincent A, Anuforo B, Btoush R, Crabtree BF. Understanding primary care physician perspectives on recommending HPV vaccination and addressing vaccine hesitancy. Human Vaccines & Immunotherapeutics. 2021. p. 1–7. 10.1080/21645515.2020.1854603 PMC8189098 33439768

[27] Mitchell K, Saraiya M, Bhatt A (2019). Increasing HPV vaccination rates through national provider partnerships. J Women's Health (Larchmt).

[28] Shapiro GK, Guichon J, Kelaher M (2017). Canadian school-based HPV vaccine programs and policy considerations. Vaccine.

[29] Feiring B, Laake I, Christiansen IK, Hansen M, Stalcrantz J, Ambur OH (2018). Substantial decline in prevalence of vaccine-type and nonvaccine-type human papillomavirus (HPV) in vaccinated and unvaccinated girls 5 years after implementing HPV vaccine in Norway. J Infect Dis.

[30] Hoeffel EM, Rastogi S, Kim MO, Shahid H (2012). The Asian population: 2010.

[31] Dorell CG, Jain N, Yankey D (2011). Validity of parent-reported vaccination status for adolescents aged 13-17 years: National Immunization Survey-Teen, 2008. Public Health Rep.

[32] Apte G, Pierre-Joseph N, Vercruysse JL, Perkins RB (2015). Could poor parental recall of HPV vaccination contribute to low vaccination rates?. Clin Pediatr (Phila).

