# Supplementary material for: Disparities in HPV vaccine knowledge and adolescent HPV vaccine uptake by parental nativity among diverse multiethnic parents in New Jersey
Source: BMC Public Health. 2022 Jan 29;22:195. doi: 10.1186/s12889-022-12573-7 (PMC8800253; doi:10.1186/s12889-022-12573-7)
Supplement: Supplementary file 1 — Additional file 1. NJ Healthy Families Survey English. [file 12889_2022_12573_MOESM1_ESM.pdf]

Rutgers Cancer Institute of New Jersey
RUTGERS HEALTH

NCI Designated Comprehensive Cancer Center

Study ID ______________

NJ Healthy Families Survey

1. Do you have an adolescent(s) in your household between 11 and 18 years old?
   ☐ No → Thank you for your time
   ☐ Yes

2. If yes, how many? ___________

3. Please provide the age and gender of each adolescent in your household below (you do not need to provide their names).

Age	Gender	Do you make the health care decisions for this/these adolescent(s)? (Yes or no)
a.		
b.		
c.		
d.		
e.		

RUTGERS | eIRB
APPROVED
IRB ID: Pro2018001695
Approval Date: 3/1/2019
Expiration Date: 10/16/2019

Study ID ________________

HPV KNOWLEDGE

4. Have you ever heard of HPV? HPV stands for Human Papillomavirus. It is not HIV, HSV, or herpes
   ☐ No → **GO TO 6**
   ☐ Yes

5. Where did you hear about HPV? Please mark all that apply.
   ☐ Health care provider/clinic
   ☐ Family and/or friends
   ☐ TV advertisements/News/TV show
   ☐ Work or school
   ☐ Newspaper/magazine
   ☐ Internet
   ☐ Some other place. Please specify:____________________________

6. Please state whether you agree or disagree with the following statements:

Do you think HPV can cause…	**Agree**	**Disagree**	**Not sure**
a. Cervical Cancer	☐	☐	☐
b. Penile Cancer	☐	☐	☐
c. Anal Cancer	☐	☐	☐
d. Oral Cancer	☐	☐	☐
e. AIDS	☐	☐	☐
g. abnormal Pap smears	☐	☐	☐

Next are some questions on your opinion about HPV.

	**Agree**	**Disagree**	**Not sure**
a. HPV is a rare infection	☐	☐	☐
b. HPV is a sexually transmitted infection (STI)	☐	☐	☐
c. HPV will go away on its own without treatment	☐	☐	☐
d. A person can have HPV for many years without knowing it or without showing signs or symptoms	☐	☐	☐
e. HPV can affect a woman's ability to get pregnant	☐	☐	☐
f. Men cannot get HPV	☐	☐	☐
g. Using condoms reduces the risk of getting HPV	☐	☐	☐
h. Having many sexual partners increases the risk of getting HPV	☐	☐	☐
i. HPV has many different types	☐	☐	☐

RUTGERS | eIRB
APPROVED
IRB ID: Pro2018001695
Approval Date: 3/1/2019
Expiration Date: 10/16/2019

Study ID ________________

HPV VACCINE KNOWLEDGE

7. A vaccine to prevent HPV infection is available and is called the HPV shot, cervical cancer vaccine, GARDASIL, or Cervarix. Before today, have you ever heard of the cervical cancer vaccine or shot?
   ☐ No
   ☐ Yes

8. How effective do you think the HPV vaccine is in preventing cervical cancer?
   ☐ Very effective
   ☐ Somewhat effective
   ☐ Not effective

9. The HPV vaccine is most effective when it is given…
   ☐ Before someone starts having sex
   ☐ After someone starts having sex
   ☐ It does not matter when the vaccine is given
   ☐ I do not know

10. Please state whether you agree or disagree with the following statements:

Statement	Agree	Disagree	Not sure
a. Getting the HPV vaccine may cause problems getting pregnant or conceiving a child later in life	☐	☐	☐
b. If my son/daughter gets the HPV vaccine, it may cause health problems in the future.	☐	☐	☐
c. My son/daughter may be more likely to think it is okay to have sex if he/she gets the HPV vaccine.	☐	☐	☐
d. HPV vaccines require more than one dose	☐	☐	☐
e. The HPV vaccines offer protection against all sexually transmitted infections	☐	☐	☐
f. The HPV vaccines are most effective if given to people who have never had sex	☐	☐	☐
g. Someone who has had the HPV vaccine cannot develop cervical cancer	☐	☐	☐
h. The HPV vaccines offer protection against most cancers	☐	☐	☐
i. One of the HPV vaccines offers protection against genital warts	☐	☐	☐
j. Girls who have had the HPV vaccine do not need a Pap smear test when they are older	☐	☐	☐
k. The HPV vaccine is only recommended for girls	☐	☐	☐

RUTGERS | eIRB
APPROVED
IRB ID: Pro2018001695
Approval Date: 3/1/2019
Expiration Date: 10/16/2019

Study ID ________________

ACCESS TO HPV VACCINE

11. When is the birthday (Month and Year only) of this son or daughter? ______(Month)__________(year)

    11a. What is the age of your adolescent son or daughter whose birthday is the closest to today's date?______years old

    Instructions: When answering the following questions please think **only** about your adolescent son or daughter whose birthday is the closest to today's date.

12. In the last 12 months, has a doctor or health care professional ever talked with you or an immediate family member about the HPV shot or vaccine?

    ☐ No

    ☐ Yes

13. In the last 12 months, has a doctor or health care professional recommended that you or someone in your immediate family get an HPV shot or vaccine?

    ☐ No → **GO to Q15**

    ☐ Yes

14. At what age did the doctor or health care professional recommend that your son/daughter should start receiving HPV shots?

    ☐ Before age 11

    ☐ 11 or 12 years of age

    ☐ 13 or 14 years of age

    ☐ 15 or 16 years of age

    ☐ 17 or 18 years of age

    ☐ After 18 years of age

    ☐ No specific age was recommended/discussed

15. Has your son/daughter ever received the HPV vaccine or HPV shot?

    ☐ No → **GO to Q17**

    ☐ Yes

RUTGERS | eIRB
APPROVED
IRB ID: Pro2018001695
Approval Date: 3/1/2019
Expiration Date: 10/16/2019

15a. If yes, where has your son/daughter received an HPV shot?

☐ Doctor's office

☐ Emergency room

☐ Health department clinic

☐ Clinic or health center

☐ Elementary/Middle/High School clinic

☐ Other nonmedical place

☐ I do not know

15b. What age did your son/daughter receive the HPV shots?_________Years old

16. How many doses has she received?

☐ One→ **GO to Q20**

☐ Two

☐ Three

16a. If more than one, how were you reminded that your son/daughter was due for the second (or third) dose? → **After answering, GO to Q20**

☐ A phone call from the provider's office

☐ Automated system – email or text

☐ Patient portal

☐ I was not reminded; I remembered

17. A vaccine to prevent the human papillomavirus or HPV infection is recommended for girls ages 11-12 and is called the cervical cancer vaccine, HPV shot, or GARDASIL®. If you had a daughter that age, would you have her get it?

☐ No

☐ Yes → **GO to Q18**

☐ I do not know

RUTGERS | eIRB
APPROVED
IRB ID: Pro2018001695
Approval Date: 3/1/2019
Expiration Date: 10/16/2019

17a. If no, what is the main reason you would not have your son/daughter get the vaccine?

☐ My son/daughter does not need the vaccine

☐ My son/daughter is not sexually active

☐ It is too expensive

☐ He/she is too old for the vaccine

☐ My son/daughter's doctor has not recommended it

☐ I am worried about the safety of the vaccine

☐ I do not know where to get it

☐ My spouse/family member is against it

☐ My son/daughter already has HPV

☐ I do not know

☐ Other, please specify: ____

18. If he/she have not already, would you be interested in your son/daughter getting the HPV vaccine?

☐ No

☐ Yes → **GO to Q19**

☐ I do not know

18a. If no, what is the main reason you would not want your son/daughter to get the vaccine?

☐ My son/daughter does not need the vaccine

☐ My son/daughter is not sexually active

☐ It is too expensive

☐ He/she is too old for the vaccine

☐ My son/daughter's doctor has not recommended it

☐ I am worried about the safety of the vaccine

☐ I do not know where to get it

☐ My spouse/family member is against it

☐ My son/daughter already has HPV

☐ I do not know

☐ Other, please specify: ____

19. If you could get the HPV vaccine free or at a low cost, would you let your son/daughter get it?

☐ No

☐Yes

☐ Don't know

RUTGERS | eIRB
APPROVED
IRB ID: Pro2018001695
Approval Date: 3/1/2019
Expiration Date: 10/16/2019

Study ID ________________

20. Has your son/daughter ever been told by a doctor or health care professional that they had a human papillomavirus or HPV infection?
    - ☐ No
    - ☐ Yes
    - ☐ I do not know

RUTGERS | eIRB
APPROVED
IRB ID: Pro2018001695
Approval Date: 3/1/2019
Expiration Date: 10/16/2019

Study ID _______________

HEALTHCARE UTILIZATION

<u>Instructions</u>: When answering the following questions please think **only** about your adolescent son or daughter whose birthday is the closest to today's date.

21. Where does your son/daughter usually go for their healthcare services?

☐ Doctor's office
☐ Emergency room
☐ Health department clinic
☐ School clinic
☐ Clinic or health center

21a. Please provide the following information about the <u>facility</u> where your son/daughter goes for their healthcare services.

Name:_________________________________ Town and City: _________________________________

21b. In the past 12 months, how often has your son/daughter gone to this facility for healthcare services?
☐ Once this year
☐ Twice this year
☐ Three or more times this year

22. What kind of health insurance does your son/daughter <u>currently</u> have?
☐ No insurance/uninsured
☐ Private health insurance or HMO purchased through my employer
☐ Private health insurance or HMO purchased by a family member or myself
☐ Medicaid
☐ Children's Health Insurance Program (CHIP)
☐ I do not know
☐ Other, please specify: _________________________________

RUTGERS | eIRB
APPROVED
IRB ID: Pro2018001695
Approval Date: 3/1/2019
Expiration Date: 10/16/2019

23. In the last 12 months, has a doctor or health care professional ever talked with you or your son/daughter about the Tdap vaccine?

☐ No

☐ Yes

☐ I do not know

24. In the last 12 months, has a doctor or health care professional recommended that your son/daughter get a Tdap vaccine?

☐ No → **GO to Q25**

☐ Yes

☐ I do not know

24a. At what age did the doctor or health care professional recommend that your son/daughter should start receiving Tdap vaccine?

☐ Before age 11

☐ 11 or 12 years of age

☐ 13 or 14 years of age

☐ 15 or 16 years of age

☐ 17 or 18 years of age

☐ After 18 years of age

☐ No specific age was recommended/discussed

☐ I do not know

25. Has your son/daughter ever received the Tdap vaccine?

☐ No

☐ Yes

☐ I do not know

26. In the last 12 months, has a doctor or health care professional ever talked with you or your son/daughter about the meningococcal vaccine?

☐ No

☐ Yes

☐ I do not know

RUTGERS | eIRB
APPROVED
IRB ID: Pro2018001695
Approval Date: 3/1/2019
Expiration Date: 10/16/2019

27. In the last 12 months, has a doctor or health care professional recommended that your son/daughter get a meningococcal vaccine?

☐ No → **GO to Q28**
☐ Yes
☐ I do not know

27a. At what age did the doctor or health care professional recommend that your son/daughter should start receiving meningococcal vaccine?

☐ Before age 11
☐ 11 or 12 years of age
☐ 13 or 14 years of age
☐ 15 or 16 years of age
☐ 17 or 18 years of age
☐ After 18 years of age
☐ No specific age was recommended/discussed
☐ I do not know

28. Has your son/daughter ever received the meningococcal vaccine?

☐ No
☐ Yes
☐ I do not know

RUTGERS | eIRB
APPROVED
IRB ID: Pro2018001695
Approval Date: 3/1/2019
Expiration Date: 10/16/2019

DEMOGRAPHICS

Instructions: We would like to know some information about **your** background. All of your responses will be confidential.

29. What is your age?___________years old

30. What is your gender identity?
☐ Male
☐ Female
☐ Non binary/Genderqueer
☐ Prefer not to answer

31. Which of the following describes your race? Please check all that apply.
☐ White
☐ Black or African American
☐ American Indian or Alaska Native
☐ Asian or Asian American
☐ Native Hawaiian/Other Pacific

32. Are you Hispanic or Latinx? Please check all that apply.
☐ No
☐ Yes
☐ Prefer not to answer
☐ Mexican, Mexican American
☐ Puerto Rican
☐ Dominican
☐ Other, please specify:________

33. If Asian or Asian American, please specify (check all that apply):
☐ Asian Indian
☐ Chinese
☐ Filipino
☐ Japanese
☐ Vietnamese
☐ Korean

RUTGERS | eIRB
APPROVED
IRB ID: Pro2018001695
Approval Date: 3/1/2019
Expiration Date: 10/16/2019

34. Were you born in the United States?

☐ No → If not, where were you born? _

☐ Yes

☐ Prefer not to answer

35. Was your son/daughter born in the United States?

☐ No → If not, where was your son/daughter born? ____

☐ Yes

☐ Prefer not to answer

36. What is your current marital status?

☐ Married or living as married

☐ Divorced

☐ Widowed

☐ Separated

☐ Single, never been married

☐ Prefer not to answer

37. What is the highest grade or level of schooling you completed?

☐ Less than 8 years

☐ 8 through 11 years

☐ High school graduate or GED

☐ Post high school training other than college (vocational or technical)

☐ Some college

☐ College graduate

☐ Graduate degree

☐ Doctorate

☐ Prefer not to answer

38. Are you currently…

☐ Employed

☐ Unemployed

☐ Homemaker

☐ Retired

☐ Student

☐ Disabled

☐ Prefer not to answer

RUTGERS | eIRB
APPROVED
IRB ID: Pro2018001695
Approval Date: 3/1/2019
Expiration Date: 10/16/2019

39. Which of the following categories best describes your household income?
   - ☐ Less than $10,000
   - ☐ $10,000 to $19,999
   - ☐ $20,000 to $29,999
   - ☐ $30,000 to $39,999
   - ☐ $40,000 to $49,999
   - ☐ $50,000 to $59,999
   - ☐ $60,000 to $69,999
   - ☐ $70,000 to $79,999
   - ☐ $80,000 to $89,999
   - ☐ $90,000 or more

40. Do you currently rent or own your home?
   - ☐ Own
   - ☐ Rent
   - ☐ Occupied without paying monetary rent
   - ☐ Prefer not to answer

41. Including yourself, how many people live in your household? ______________

42. What kind of health insurance do you currently have?
   - ☐ No insurance/uninsured
   - ☐ Private health insurance or HMO purchased through my employer
   - ☐ Private health insurance or HMO purchased by a family member or myself
   - ☐ Medicaid
   - ☐ Medicare
   - ☐ I do not know
   - ☐ Other, please specify: _____________________________________

RUTGERS | eIRB
APPROVED
IRB ID: Pro2018001695
Approval Date: 3/1/2019
Expiration Date: 10/16/2019
